# Preparation and *in vitro* and *in vivo* evaluations of 10-hydroxycamptothecin liposomes modified with stearyl glycyrrhetinate

**DOI:** 10.1080/10717544.2019.1636422

**Published:** 2019-07-02

**Authors:** Ting Zhou, Xin Tang, Wei Zhang, Jianfang Feng, Wei Wu

**Affiliations:** a School of Pharmacy, Guilin Medical University, Guilin, P.R. China;; b School of Public Health, Guilin Medical University, Guilin, P.R. China;; c School of Pharmacy, Guangxi University of Chinese Medicine, Nanning, P.R. China

**Keywords:** 10-hydroxycamptothecin, stearyl glycyrrhetinate, liposomes, pharmacokinetics, biodistribution

## Abstract

10-Hydroxycamptothecin (HCPT) liposomes surface modified with stearyl glycyrrhetinate (SG) were prepared by the film dispersion method. Characterization of the liposomes, including drug release *in vitro*, pharmacokinetics and tissue distribution, was done. HCPT in plasma and tissues was determined by high-performance liquid chromatography (HPLC). Compared with the conventional HCPT-liposomes and commercially available hydroxycamptothecin injection (HCPT Inject), pharmacokinetic parameters indicated that SG-HCPT-liposomes had better bioavailability. Regarding tissue distribution, the concentration of HCPT loaded by SG modified liposomes in the liver was higher than other tissues and the risk to the kidney was lower than HCPT-liposomes and HCPT Inject. These results support the hypothesis that the HCPT-liposomes modified with SG show enhanced liver-targeting through the glycyrrhetinic acid (GA) receptor to be an efficient drug carrier, which may help to improve therapeutic methods for hepatic diseases in the future.

## Introduction

10-Hydroxycamptothecin is a promising derivative of camptothecin, which is a natural quinoline alkaloid extracted and isolated from the bark and stems of *Camptotheca acuminate* (Shi et al., 2010; Chen et al., 2015a). HCPT has been shown to be more active and less toxic than camptothecin (Zhu et al., 2013). HCPT mainly acts on DNA synthesis during the S phase (Yang et al., 2011). It is thought to interfere with DNA replication and transcription by inhibiting the activity of topoisomerase I (Topo I), leading to the apoptosis of tumor cells (Du et al., 2017). As an anticancer drug, it has attracted considerable attention due to multiple antitumor activities against hepatoma, bladder cancer, gastric cancer, colorectal cancer, leukemia and so forth (Sayari et al., 2014; Cardillo et al., 2015; Min et al., 2018; Ye et al., 2018a). Although HCPT is active against various cancers, its major drawbacks are poor solubility and a short half-life (Ye et al., 2018b). Clinical HCPT sodium injection causes some adverse reactions, for example, myelosuppression, hemorrhagic cystitis, diarrhea, nausea, vomiting, and dermatitis, which are obstacles to wider clinical application of HCPT (Wang et al., 2013). To overcome these problems, an emerging application is to use liposomes as vehicles for HCPT delivery.

Liposomes, which are composed of two layers, are widely accepted as a drug delivery system (Liu et al., 2016a). Compared to free drugs, liposomal formulations achieve more drug accumulation in the tumor region through an enhanced permeability and retention (EPR) effect, prolonged blood circulation time, reduced drug toxicity and increased therapeutic efficacy (Maruyama, 2011; Corvo et al., 2016). Nevertheless, the passive targeting effect of liposomes cannot guarantee increased cellular uptake of the drug (Li et al., 2015; Liu et al., 2016b). In recent years, aiming to improve the targeting and stability of liposomes, many scholars have studied surface modification of liposome membranes (Qi et al., 2015; Xie et al., 2015; Yang et al., 2015; Zhu et al., 2015; Xie et al., 2016; Liu et al., 2017). Glycyrrhetinic acid (GA) is one of the main bioactive compounds extracted from licorice, which has been used to treat hepatic disease (Tian et al., 2012; Zhang et al., 2012; Darvishi et al., 2015; Jing et al., 2017a). Stearyl glycyrrhetinate (SG), the stearyl ester of 18-β-glycyrrhetinic acid, is a derivative of GA. It has been demonstrated that GA and its derivatives may be used as ligands targeting the liver (Radwan et al., 2016; Zhu et al., 2018). Because it has been shown that abundant receptors for GA exist on liver cell membranes, several recent research efforts have attempted to utilize GA as a targeting ligand for hepatocyte-targeting (He et al., 2010; Huang et al., 2011; Shi et al., 2012; He et al., 2014; Zhang et al., 2015; Chen et al., 2015b).

In the present investigation, HCPT as a model antitumor drug was incorporated into liposomes. HCPT-liposomes and HCPT-liposomes conjugated with SG were prepared by the film-dispersion method. Their physicochemical characteristics such as size distribution, morphology and surface charge were investigated. The *in vitro* drug release as well as *in vivo* plasma pharmacokinetics and tissue distribution studies of SG-HCPT-liposomes were evaluated compared to the unmodified HCPT-liposomes and HCPT injection. We hope that our studies will be helpful in providing a reference for clinical application.

## Materials and methods

### Materials

10-Hydroxycamptothecin bulk drugs (HCPT, purity ≥98%), soybean phospholipids (SPC, purity >98%), cholesterol (Chol, purity >95%), hydroxycamptothecin standard (98%, Lot No.: H1524105), and camptothecin (CPT, purity ≥98%, Lot No.: H1810045) as the internal standard (I.S.) were purchased from Aladdin Industrial Corporation (Shanghai, China). Hydroxycamptothecin for injection was obtained from Shenzhen Main Luck Pharmaceuticals Inc. (Shenzhen, China). Stearyl glycyrrhetinate (SG, purity ≥95%) was purchased from Sigma-Aldrich (Japan). Methanol was of HPLC grade. All other reagents were of analytical grade, and purified deionized water was used throughout.

Female SD rats (220–250 g) and KM mice (20–30 g) were purchased from Hunan Silaikejingda Laboratory Animal Co., Ltd., Hunan, China (Certificate No. SCXK 2016–0002). Experiments were conducted in accordance with the guidelines issued by the State Food and Drug Administration (SFDA of China). The animals were housed and cared for in accordance with the guidelines established by the National Science Council of the Republic of China.

### Preparation of hydroxycamptothecin liposomes (HCPT-liposomes) and stearyl glycyrrhetinate-modified liposomes (SG-HCPT-liposomes)

The liposomes were prepared by the film dispersion method (Chen et al., 2015b). A mixture of SPC, Chol, and HCPT (130, 40, and 7 mg) was dissolved in 10 mL of chloroform solution, and then the solvent was evaporated by a rotator evaporator (RE-52CS-1; ShangHai Yarong Biochemistry Instrument Factory, ShangHai) under vacuum at 60 °C to form a thin film on the inner walls of the round-bottom flask. The film was vacuum-dried to remove residual organic solvent for 2 h. The lipid film was finally hydrated with 5 mL of PBS (pH 6.8) at 50 °C for 2 h. Liposomes were sonicated for 5 min at 300 W in an ice bath using an ultrasonic cell disruptor (SCIENTZ-IID, Scientz, China), and filtrated through 0.22-µm membranes to sterilize the final formulations and also remove aggregates. Finally, the liposomes were stored at 4 °C for further characterization.

SG-HCPT-liposomes were prepared in a similar way with an additive of 3.9 mg of stearyl glycyrrhetinate (3% weight of SPC).

### Characterization of the liposomes

The morphology of HCPT-liposomes and SG-HCPT-liposomes was investigated by transmission electron microscopy (TEM, HT7700, HITACHI, Japan). Samples were diluted 5-fold, placed on copper grids, and negatively stained with 2% phosphotungstic acid before further analysis. The Z-average size, zeta potential, and polydispersity index (PDI) were determined using a Malvern Zetasizer Nano-S90 analyzer (Malvern Instruments, Malvern, UK). Free drugs were separated from the liposomes using Sephadex G-50 gel for measurement of entrapment efficiency. Briefly, 200 µL of liposomes was loaded onto a Sephadex G-50 gel column and eluted at 0.5 mL/min with phosphate buffer (pH 6.8), followed by separation of the liposomes and free drugs. The encapsulated liposomes were collected, and the concentrations of HCPT was quantified using high-performance liquid chromatography (HPLC; 20 A, SHIMADZU, Japan) at 370 nm with a reversed phase InertSustain-C_18_ analytical column (250 mm × 4.6 mm, 5 μm). The mobile phase was a mixture of methanol–water at 55:45 (v/v). The flow rate was 1 mL/min. The injection volume was 20 μL. The percent entrapment efficiency of HCPT-liposome and SG-HCPT-liposome formulations was calculated using Equation (1), and the percent drug loading of HCPT-liposome and SG-HCPT-liposome formulations was calculated using Equation (2).

(1) EE%=entrapped drug/initial drug×100%

(2) DL%=entrapped drug/weight of carrier×100%

where EE% is the percent entrapment efficiency, DL% is the percent drug loading, entrapped drug is the amount of drug encapsulated in the liposomes, initial drug is the amount of drug added to the system, and weight of carrier is the amount of the liposomes used (all in mg).

### *In vitro* drug release

*In vitro* release of drug from HCPT Inject, HCPT-liposome and SG-HCPT-liposome formulations was analyzed by the dialysis bag method (Chen et al., 2015b). Briefly, 1 mL of HCPT Inject, HCPT-liposomes and SG-HCPT-liposomes containing 1.4 mg of drug was placed in a dialysis bag with a molecular weight cutoff of 8 kDa-12 kDa. Then, the dialysis bag was suspended in 200 mL of PBS (pH 7.4) under sinking conditions. The beaker was left on a magnetic stirrer (200 rpm) for 48 h at 37 °C. At predetermined time intervals, aliquots of the medium were withdrawn from the beaker and replaced with an equal volume of fresh PBS solution. The amount of drug released into the medium was determined by the aforementioned HPLC method. The accumulated release of HCPT Inject, HCPT-liposomes and SG-HCPT-liposomes was calculated by the following equation (*n* = 3):

$$\text{Drug release percentage }\left(\%\right)={\text{W}}_{\text{release}}/{\text{W}}_{\text{total}}\times 100\%$$

### EE, LD and particle size stability *in vitro*

Liposome stability under *in vitro* storage conditions is an important criterion for both *in vitro* and *in vivo* biomedical applications. To investigate the EE, LD, particle size and PDI stability of HCPT-liposomes and SG-HCPT-liposomes in PBS (pH 6.8) at 4 °C in the dark, two batches of liposomes (HCPT-liposomes and SG-HCPT-liposomes) were assessed at various time points (0, 1, 7, 15, and 30 days) by the methods described in the previous sections.

### Pharmacokinetic studies

Fifteen SD rats were randomly divided into three groups with five mice in each group. HCPT Inject, HCPT-liposomes and SG-HCPT-liposomes were injected intravenously into the tail vein of the mice (8 mg/kg). Blood samples (0.5 mL) were drawn from the socket at predetermined intervals of 0.033, 0.083, 0.167, 0.5, 1, 2, 4, 6, 8 and 12 hours postdose into heparinized tubes. Blood was immediately centrifuged at 12,000 rpm for 10 minutes. Plasma samples were obtained and stored in a −20 °C freezer and analyzed within 3 days.

### Biodistribution studies

One hundred twenty KM mice were divided into three groups at random with 40 mice in each group and given a dose of 8 mg/kg HCPT Inject, HCPT-Lip or SG-HCPT-Lip by tail vein injection. At 0.167, 0.5, 1, 2, 4, 6, 8 and 12 hours after drug injection, each animal (*n* = 5 for each time point) was subsequently sacrificed by cervical dislocation, and the major organs including the heart, liver, spleen, lung, and kidney were collected. Tissue samples were washed in ice-cold saline, blotted with a paper towel to remove excess fluid, and the organs were weighed and homogenized in 0.5 mL of saline solution per 100 mg of tissue. The samples were stored at −20 °C and analyzed in 3 days.

### HPLC analysis of HCPT in plasma and tissue samples

To determine the amount of drug accumulation, approximately 20 µL of CPT (50 µg/mL) solution (IS) and 10 μL of glacial acetic acid were added to 200 µL of plasma or homogenate, and the mixture was incubated in the dark for 2 h after vortex mixing. Then 1 mL of methanol solution was added, and the mixture was vortexed for 3 min, followed by centrifugation at 13,000 rpm for 10 min. Finally, 20 µL of supernatant was injected into the HPLC system for analysis after being filtered through a 0.22-µm microfiltration membrane. Standard curves were constructed by plotting the ratio of HCPT to internal standard CPT peak areas as a function of known concentration.

### Statistical analysis

Data are expressed as the mean ± standard deviation (SD). *p* < .05 was considered statistically significant as determined by Student’s unpaired t-test using the SPSS software (version 17.0, SPSS Inc., Chicago, IL). The pharmacokinetic parameters of HCPT were analyzed using 3p97 software by compartmental analysis.

## Results and discussion

### Characterization of the HCPT-liposomes and SG-HCPT-liposomes

**Figure 1.**
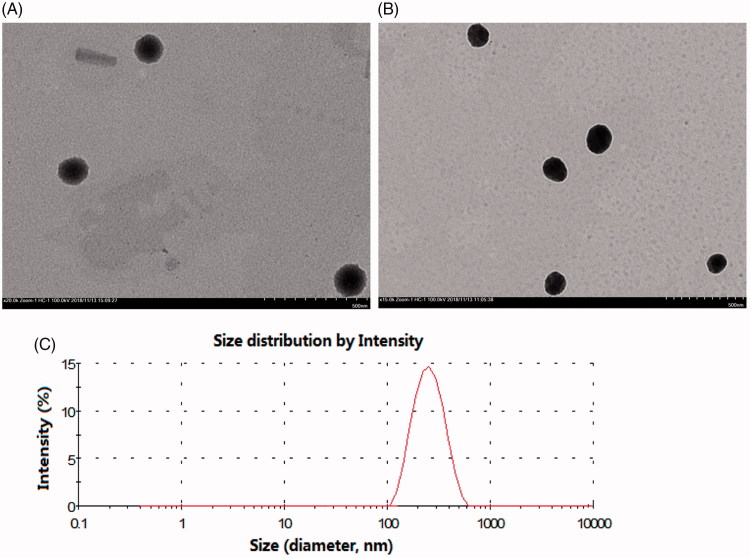
Size distribution and transmission electron microscope (TEM) photographs of liposomes. (A) TEM image of HCPT-liposomes. (B) TEM image of SG-HCPT-liposomes. (C) Size distribution of SG-HCPT-liposomes.

As shown in Figure 1, the morphology of the two liposomes was spherical with a uniform size as evaluated under a transmission electron microscope. The particle size, zeta potential, PDI, EE and LD of the liposomes are shown in Table S1. The average sizes of the HCPT-liposomes and SG-HCPT-liposomes were approximately 157 and 168 nm, respectively. It has been reported that liposomes ranging from 100–200 nm in diameter can avoid physical clearance and significantly accumulate in the tissue due to an enhanced permeability and retention (EPR) effect (Jing et al., 2017b). The PDI of liposomes was reasonably low (<0.25). Both liposomes were negatively charged, with zeta potentials of -21.2 and -22.8 mV, respectively, which is beneficial for liposome stability (Nayak et al., 2017). For the prepared liposomes, the encapsulation of HCPT liposomes was 75.6%, and drug loading was 2.87%, whereas the encapsulation of SG-HCPT-liposomes was 78.9%, and drug loading was 3.06%. The EE% values of both were higher than 75%. The results indicated that no significant variations occurred following the addition of SG to the liposomes, and the incorporation of SG did not affect the EE or destroy the structure of the liposomes.

### *In vitro* release

**Figure 2.**
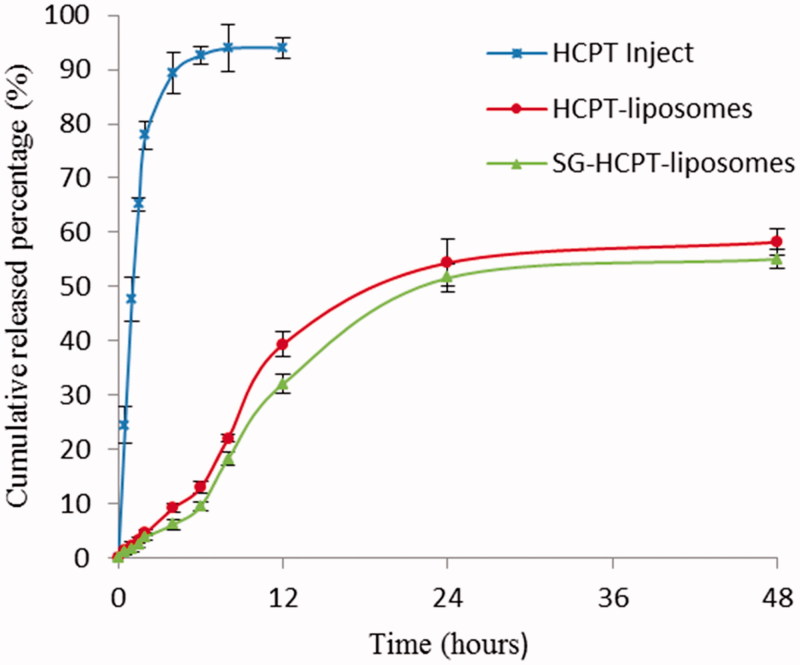
*In vitro* release profiles (37 °C, PBS, pH 7.4, *n* = 3).

The release profiles of HCPT Inject, HCPT-liposomes and SG-HCPT-liposomes are shown in Figure 2. The cumulative release of liposomes within 12 h was significantly lower than that of commercially available HCPT. Over 90% of the commercially available HCPT was released in approximately 6 hours, whereas the cumulative release of HCPT-liposomes and SG-HCPT-liposomes within 48 h was 58% and 55%, respectively.

*In vitro* data showed that the HCPT release profile was prominently prolonged by liposomal encapsulation in PBS solution compared with commercially available HCPT. These results were favorable for further *in vivo* application of the system. However, no significant differences were observed in the drug release characteristics between HCPT-liposomes and SG-HCPT-liposomes.

### Stability *in vitro*

For stability studies, the liposomes could be dispersed well by light shaking, and the appearance did not show obvious change. The particle size of the liposomes observed 1 month after the date of manufacture ranged from 157 to 166 nm in the HCPT liposomes and from 168 to 175 nm in the SG-HCPT-liposomes. In addition, the EE% ranged from 75.6% to 72.8% in the HCPT-liposomes and from 78.9% to 76.2% in the SG-HCPT-liposomes (Table S2). The particle size and EE values were not significantly different between the two liposomes, indicating that the formulations were stable for 1 month at 4 °C.

### Pharmacokinetics studies

**Figure 3.**
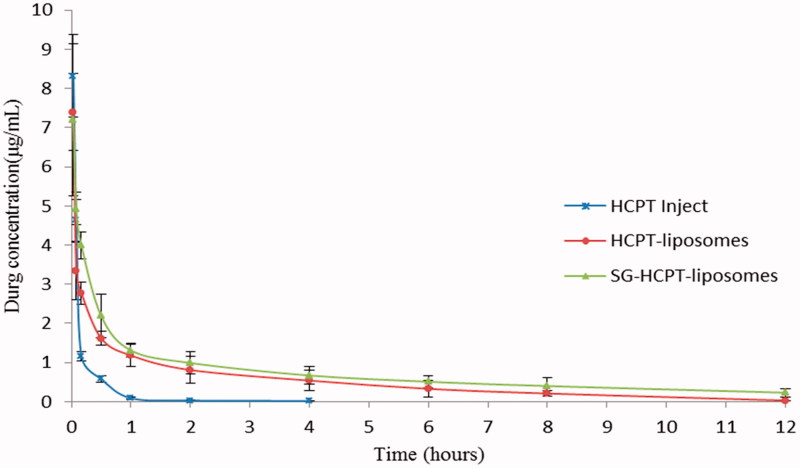
The concentration–time curves of HCPT in plasma of mice given HCPT Inject, HCPT-liposomes and SG-HCPT-liposomes after tail vein injection.

The mean concentration-time curves of HCPT Inject, HCPT-liposomes and SG-HCPT-liposomes in plasma are shown in Figure 3, and the main pharmacokinetic parameters of HCPT in plasma are summarized in Table 1. The results showed that the plasma concentration-time curves of HCPT-liposomes and SG-HCPT-liposomes were consistent with the three-compartment model, whereas HCPT Inject fit best with the two-compartment model. HCPT Inject had a short elimination half-life and a rapid clearance rate from blood circulation. The maximum concentration (*C*_max_) of HCPT Inject occurred within 2 minutes, and then the concentration stepped down quickly. The concentration was near 0 after 4 hours. The *C*_max_ of HCPT in plasma of the two liposomes was lower than the HCPT Inject, but the elimination half-life time (*t*_1/2_), the area under the curve (AUC) and the mean retention time (MRT) were much longer than HCPT Inject. This means that the two liposomes have a longer circulation and better bioavailability than HCPT Inject. The *t*_1/2_ of HCPT in the SG-liposomes (250.51 min) and liposomes (173.53 min) was 12.5 and 7.6 times longer, respectively, than that of HCPT Inject (20.07 min). The AUC of HCPT of the SG-liposomes (585.19 µg·min/mL) and liposomes (414.86 µg·min/mL) was 7.7 and 5.4 times higher, respectively, than that of HCPT Inject (76.45 µg·min/mL). The MRT of HCPT in the SG-liposomes (251.44 min) and liposomes (174.99 min) was 13.1 and 9.1 times longer, respectively, than that of HCPT Inject (19.21 min). The CL of HCPT-liposomes and SG-HCPT-liposomes was 19.28 and 13.67 mL/kg/min lower, respectively, than that of the HCPT Inject (104.13 mL/kg/min). These results can be attributed to the protection of the lipid bilayer membranes and slow drug release from liposomes, which is consistent with the results of the *in vitro* release study (Luo et al., 2016). These confirm that the SG-HCPT-liposomes have comparatively better pharmacokinetic profiles than the HCPT-liposomes and HCPT Inject.

**Table 1.** Pharmacokinetic parameters.

Parameter	HCPT Inject	HCPT-liposomes	SG-HCPT-liposomes
Compartment number	Two compartments	Three compartments	Three compartments
C_max_ (µg/mL)	8.32 ± 1.05	7.39 ± 0.99*	7.16 ± 1.94**
*t*_1/2_ (min)	21.07 ± 4.88	173.53 ± 16.22**	250.51 ± 13.49**^,#^
AUC_0-_*_t_* (µg·min/mL)	76.45 ± 6.39	414.86 ± 19.54**	585.19 ± 26.82**^,##^
MRT (min)	19.21 ± 4.97	174.99 ± 24.67**	251.44 ± 18.36**^,##^
CL (mL/kg /min)	104.13 ± 19.45	19.28 ± 3.73*	13.67 ± 3.67*

*p < .05 vs HCPT Inject, **p < .01 vs HCPT Inject.

^#^*p* < .05 vs HCPT-liposomes, ^##^*p* < .01 vs HCPT-liposomes.

HCPT: Hydroxycamptothecin; SG: Stearyl glycyrrhetinate; C_max_: the maximum concentration; *t*_1/2_: half-life time; AUC_0–_*_t_*: area under the drug concentration-time curve values (from 0 to time t); MRT: mean residence time; CL: clearance.

### Tissue distribution studies

**Figure 4.**
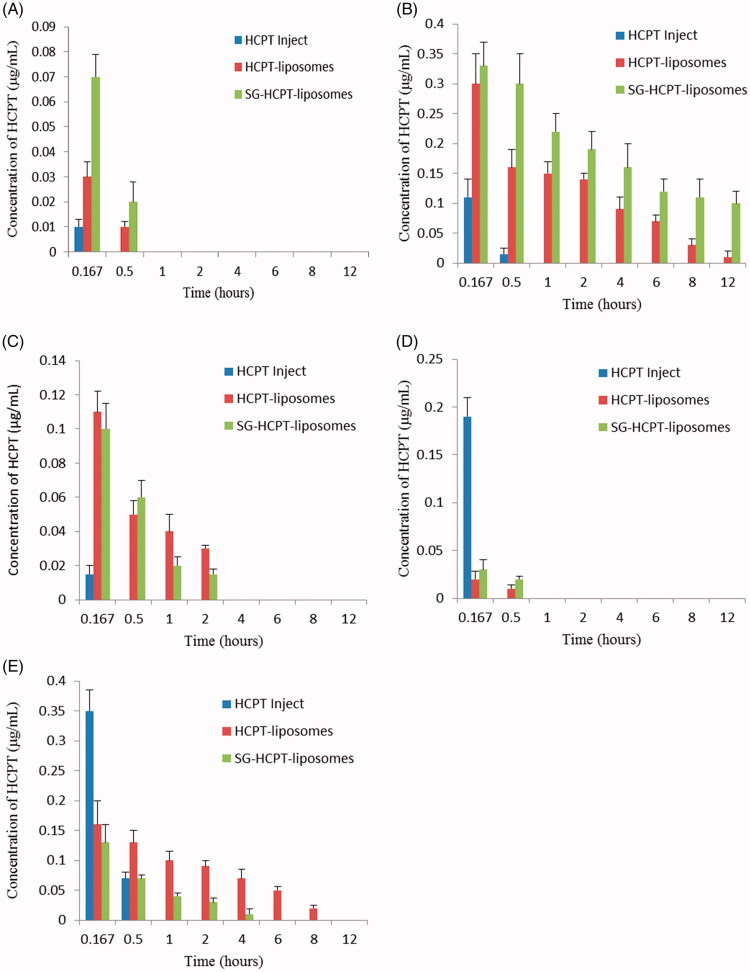
Concentration of HCPT in heart (A), liver (B), spleen (C), lung (D), and kidney (E) of mice at various time points after i.v. administration.

Figure 4 presents the results of the distribution in different organs obtained at 0.167, 0.5, 1, 2, 4, 6, 8 and 12 hours after injection of HCPT preparations into mouse tail veins. HCPT was widely and rapidly distributed into all the assessed organs with the highest concentration following intravenous administration of HCPT Inject, HCPT-liposomes, or SG-HCPT-liposomes at 10 min, as the first time point of detection. The two liposomes were mainly distributed in the liver, kidney, and spleen, followed by the heart and lung. HCPT-liposomes and SG-HCPT-liposomes experienced a rapid clearance from the heart and lung and were not detectable after 0.5 h. HCPT Inject was present in particularly high levels in the lung and kidney at 10 min, where HCPT was rapidly excreted, and were not detectable after 0.5 h. HCPT encapsulated in unmodified liposomes was also relatively high, likely due to passive targeting to the liver, spleen and kidney tissues, which are rich in reticular endothelia. However, at various times, the concentration of HCPT released from SG-HCPT-liposomes in the liver was highest, higher than in the other organs, indicating that the liposomes can deliver the drug SG rapidly to the liver after intravenous administration. The concentrations of HCPT released from HCPT-liposomes and SG-HCPT-liposomes in the spleen were much lower than in the liver and kidney and were near 0 after 2–4 hours. HCPT-liposomes retained the highest HCPT distribution in the kidney compared with the other two formulations 0.5 h after administration, which was augmented to 8 h. A negligible amount of HCPT-loaded SG-HCPT-liposomes was found in the kidney 2–4 hours after drug administration, which conferred protective effects of SG in lowering drug-associated kidney toxicity. SG-modified liposomes are biocompatible carriers that reduce the potential toxicity of HCPT, enhance its liver-targeting activity and prolong its retention time through the GA receptor. The residence time of HCPT delivered by liposomes was significantly higher than that of HCPT Inject and was augmented at 12 h for both HCPT-liposomes and SG-HCPT-liposomes.

## Conclusion

The current research describes a novel method using HCPT-loaded SG-modified liposomes compared to passively targeted HCPT-liposomes as a drug-delivery system. The prepared HCPT-liposomes and SG-HCPT-liposomes obtained by the film dispersion method had a similar entrapment efficiency and drug loading, with relatively uniform sizes and PDI below 0.25, indicating that the addition of SG had no significant effect on the physical properties of the liposomes. The liposomes exhibited excellent stability and showed sustained drug release, which are favorable characteristics for pharmaceutical nanomedicine applications. *In vivo*, the pharmacokinetics of SG-HCPT-liposomes demonstrated a prolonged circulation time compared with the commercially available hydroxycamptothecin injection, and the increased area under the curve of the HCPT-liposomes and SG-HCPT-liposomes demonstrated higher absorption. Also, we demonstrated through a biodistribution study that SG-HCPT-liposomes were effectively selected by the liver, reducing the nephrotoxicity and enhancing the safety compared with the commercially available hydroxycamptothecin injection and conventional HCPT-liposomes. This will make it possible to improve liver disease treatments. This research provides a basis for evaluating the clinical potential of SG-HCPT-liposomes.

## Supplementary Material

Supplemental Material
