# Supplementary material for: Preparation and in vitro and in vivo evaluations of 10-hydroxycamptothecin liposomes modified with stearyl glycyrrhetinate
Source: Drug Deliv. 2019 Jul 2;26(1):673–9. doi: 10.1080/10717544.2019.1636422 (PMC6610527; doi:10.1080/10717544.2019.1636422)
Supplement: Supplemental Material [file IDRD_A_1636422_SM4731.doc]

**SUPPLEMENTARY MATERIAL**

**Preparation and *in vitro* and *in vivo* evaluations of 10-hydroxycamptothecin liposomes modified with stearyl glycyrrhetinate**

Ting Zhou^a*^, Xin Tang^b*^, Wei Zhang^a^, Jianfang Feng^c^, Wei Wu^a^

^a^ Shool of Pharmacy, Guilin Medical University, Guilin 541004, P.R. China

^b^ Shool of Public Health, Guilin Medical University, Guilin 541004, P.R. China

^c^ Shool of Pharmacy, Guangxi University of Chinese Medicine, Nanning 530200, P.R. China

^*^ They contributed equally to this work.

Corresponding authors: Wei Wu, Shool of Pharmacy, Guilin Medical University, Huancheng North 2^nd^109, Guilin 541004, P.R. China. Email: wuwei@glmc.edu.cn

**Abstract**

10-Hydroxycamptothecin (HCPT) liposomes surface modified with stearyl glycyrrhetinate (SG) were prepared by the film dispersion method. Characterization of the liposomes, including drug release *in vitro*, pharmacokinetics and tissue distribution, was done. HCPT in plasma and tissues was determined by high-performance liquid chromatography (HPLC). Compared with the conventional HCPT-liposomes and commercially available hydroxycamptothecin injection (HCPT Inject), pharmacokinetic parameters indicated that SG-HCPT-liposomes had better bioavailability. Regarding tissue distribution, the concentration of HCPT loaded by SG modified liposomes in the liver was higher than other tissues and the risk to the kidney was lower than HCPT-liposomes and HCPT Inject. These results support the hypothesis that the HCPT-liposomes modified with SG show enhanced liver-targeting through the glycyrrhetinic acid (GA) receptor to be an efficient drug carrier, which may help to improve therapeutic methods for hepatic diseases in the future.

**Keywords:** 10-hydroxycamptothecin; stearyl glycyrrhetinate; liposomes; pharmacokinetics; biodistribution


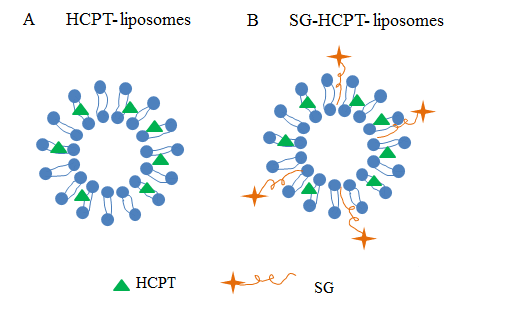


Figure S1. HCPT loaded in various liposomes.

**Abbreviations:** HCPT, Hydroxycamptothecin; SG, Stearyl glycyrrhetinate.

Table S1. Characterization of the liposomes (n=3).

| Sample | Z-average size  (nm) | Zeta potential (mV) | PDI | EE  (%) | DL  (%) |
| --- | --- | --- | --- | --- | --- |
| HCPT-liposomes | 157.2±2.6 | -21.2±1.5 | 0.231±0.07 | 75.6±2.3 | 2.87±0.17 |
| SG-HCPT-liposomes | 168.1±3.5 | -22.8±2.2 | 0.246±0.04 | 78.9±1.9 | 3.06±0.25 |

**Abbreviations:** SG, Stearyl glycyrrhetinate; HCPT, Hydroxycamptothecin; PDI, Polydispersity index; EE, Entrapment efficiency; DL, Drug loading.

Table S2. Stability data of liposomes (n=3).

| Time (days) | HCPT-liposomes | | SG-HCPT-liposomes | |
| --- | --- | --- | --- | --- |
|  | Z-average size  (nm) | EE  (%) | Z-average size (nm) | EE  (%) |
| 7 | 158.8±2.0 | 75.1±1.3 | 161.4±2.7 | 78.0±2.6 |
| 15 | 162.3±3.2 | 73.7±1.8 | 173.3±1.1 | 77.4±1.9 |
| 30 | 166.0±2.7 | 72.8±0.9 | 174.5±2.3 | 76.2±1.4 |
